# PHP.B/eB Vectors Bring New Successes to Gene Therapy for Brain Diseases

**DOI:** 10.3389/fbioe.2020.582979

**Published:** 2020-10-15

**Authors:** Robin Reynaud-Dulaurier, Michael Decressac

**Affiliations:** Univ. Grenoble Alpes, Inserm, U1216, Grenoble Institut des Neurosciences, Grenoble, France

**Keywords:** gene therapy (GT), rare disease (RD), viral vector, CNS—central nervous system, blood-brain barrier

Rare genetic diseases collectively affect around 300 million people worldwide and a staggering 74% of them are childhood-onset conditions with neurological manifestations (Lee et al., 2020). Correction or replacement of malfunctioning genes using viral vectors showed outstanding pre-clinical and clinical benefits for inherited pediatric diseases, in particular those for which non-cell autonomous mechanisms such as cross-correction is effective (i.e., X-linked adrenoleukodystrophy or Batten's disease) (Naldini, 2015; Aubourg, 2016).

However, this field still faces important challenges in particular for conditions caused by genes encoding for non-secreted proteins and whose dysfunction affect large regions of the brain that cannot be covered by intra-cerebral delivery. This includes a plethora of rare genetic conditions such as mitochondrial diseases, lysosomal storage diseases, polyglutamine diseases, and neuromuscular diseases.

Gene delivery to the brain via systemic injection can be successfully achieved during a short period of time after birth where the blood-brain barrier (BBB) remains permeable. This entails establishing a diagnosis within this privileged time-window. Unfortunately, despite being early-onset diseases, many pediatric conditions manifest the first clinical signs when the BBB is no longer penetrable by viral vectors. Because of the limitations of our current toolbox, alternative strategies had to be designed in order to allow transgene delivery to the central nervous system (CNS) when the BBB has acquired its selective permeability. Methods to engineer the viral capsid such as peptide insertion, chemical modifications, shuffled genome libraries, directed evolution, or rationally targeted mutagenesis led to the generation of tenth of artificial variants among which some have been designed with the purpose to cross more efficiently the BBB when delivered into the bloodstream (Castle et al., 2016).

By combining the insertion of randomized sequences in a lox sites-containing *cap* gene with multiple selection rounds in Cre transgenic mice, Deverman and colleagues created series of brain-penetrating adeno-associated viral (AAV) vector variants. One of these, namely the AAV-PHP.B vector, induced a transgene expression in the CNS at least 40-fold greater than the parental AAV9 capsid when injected intravenously, and it retained a cellular tropism for peripheral organs (Deverman et al., 2016). In a follow-up study, the same group further evolved the PHP.B variant for better neuronal transduction. By applying the same Cre recombinase-based AAV target evolution strategy to the PHP.B capsid, they isolated the AAV-PHP.eB variant that showed enhanced CNS infectivity, enabling the reduction of the viral load delivered intravenously (Chan et al., 2017).

The generation of these novel brain-penetrant viral vectors opened a unique opportunity to overcome the obstacle that represents the BBB in the development of gene therapy for conditions with CNS pathology. Recent reports show that this idea was indeed quickly picked up by several groups and applied to a wide range of genetic diseases. They confirmed that the engineered AAV-PHP.B and AAV-PHP.eB variants efficiently transduced the CNS following injection in the vascular milieu. These tools were then used to restore the expression of defectives genes in several mouse models of mitochondrial diseases (i.e., mitochondrial hyper-fusion, and Leigh syndrome), cytoskeleton dysfunction (i.e., CDKL5 deficiency disorder), Rett syndrome and lysosomal disease (i.e., Pompe disease and Sandhoff disease). In all of these animal models, reinstatement of gene expression in the CNS and peripheral organs afforded robust therapeutic effects. The broad correction of pathological signs resulted in behavioral improvements and increased lifespan (Table 1). Notably, for some of these severe conditions, the use of these brain-penetrant AAV vectors provided the first demonstration that their respective pathological phenotypes can be rescued by gene therapy. This is for instance the case for the *Ndufs4*^−/−^ mouse model of Leigh syndrome for which previous attempts of gene replacement therapy using an AAV9-Ndusf4 vector in neonates did not show significant effects (Di Meo et al., 2017). In addition, the *in vivo* studies listed in Table 1 have been performed in KO mice which is a paradigm that does not accurately mimic the genetic defects observed in patients. Mutations often result in the synthesis of a dysfunctional protein that can exert a dominant negative effect potentially hampering the therapeutic efficacy of the transgene. This question started to be addressed by Gao and colleagues who extended their findings in iPSC-derived neurons from a patient with CDKL5 disorder and confirmed the phenotypic improvement (Gao et al., 2020).

**Table 1:** Listing the preclinical studies of gene replacement therapies using the PHP.B or PHP.eB vectors in mouse models of rare genetic diseases with CNS pathology.

**Disease (animal model)**	**Vector and strategy**	**Biological outcomes**	**References**
Mitochondria hyper-fusion (*Slc25a46*^−/−^ mice)	AAV-PHP.B-Slc25a46 2 × 10^11^gc/g, Temporal vein at P1-2	Restored gene expression Increased lifespan Rescued brain, eye, and peripheral nerve pathology	Yang et al., 2020
Cdkl5 deficiency disorder (*Cdlk5*^−/−^ mice)	AAV-PHP.B-Cdkl5 10^12^ vg/mouse Intra-jugular at P28-30	Restored gene expression Improved behavioral phenotype Rescued brain pathology	Gao et al., 2020
Leigh syndrome (*Ndufs4*^−/−^ mice)	AAV-PHP.B-Ndufs4 10^12^ vg/mouse Retro-orbital sinus at P28	Restored gene expression Increased lifespan Improved behavioral phenotype Rescued brain, eye, and heart pathology	Reynaud-Dulaurier et al., 2020
Leigh syndrome (*Ndufs4*^−/−^ mice)	AAV-PHP.B-Ndufs4 1 × 10^12^–2 × 10^12^ vg/mouse Tail vein at P26-28	Restored gene expression Increased lifespan Rescued brain pathology	Silva-Pinheiro et al., 2020
Rett syndrome (*Mecp2*^−/−^ mice)	AAV-PHP.eB-iMecp2 1 × 10^9^–1 × 10^12^ vg/mouse Tail vein at P28	Restored gene expression Increased lifespan Improved locomotor function Rescued brain pathology	Luoni et al., 2020
Pompe disease (*Gaa*^−/−^ mice)	AAV-PHP.B-GAA 5 × 10^12^ vg/kg Retro-orbital sinus at P14	Restored gene expression Rescued brain and muscle pathology	Lim et al., 2019
Sandhoff disease (*Hexb*^−/−^ mice)	AAV-PHP.B-HEXA/HEXB 3 × 10^11^–1 × 10^12^ Tail vein at P28-42	Restored gene expression Increased lifespan Improved behavioral phenotype Rescued brain and liver pathology	Lahey et al., 2020

One can anticipate that this first set of pre-clinical studies will pave the way for similar strategies in other models of severe inherited conditions for which no curative options are currently available. In addition, the positive outcomes observed in these models of fatal neurological disorders make the PHP variants attractive tools for other brain conditions with late-onset. It this line, other groups have already extended the therapeutic repertoire of these new viral vectors by successfully tested them in models of Parkinson's and Alzheimer's disease (Morabito et al., 2017; Jin et al., 2020). Targeted delivery of AAV-PHP.B vectors also provided benefits in mouse models of deafness and retinitis pigmentosa (Giannelli et al., 2018; Gyorgy et al., 2019).

In parallel to these gene therapy studies, other research groups examined the versatility of the PHP variants. Since these artificial serotypes were isolated after several rounds of *in vivo* selection in C57BL6 mice, the brain-penetrant property of the AAV-PHP.B vector was tested in other genetic backgrounds. These experiments revealed that its ability to cross the BBB is strain-dependent and genetic approaches identified the lymphocyte antigen 6 complex locus A (Ly6a), a glycolsylphosphatidylinositol-anchored protein expressed by endothelial cells, as a key determinant for this differential efficacy (Hordeaux et al., 2018, 2019; Challis et al., 2019; Huang et al., 2019; Matsuzaki et al., 2019; Batista et al., 2020). Moreover, when tested in non-human primates, the AAV-PHP.B vector showed lower transduction efficiency than that observed in mice, highlighting inter-species variability in the BBB penetrance of this novel AAV serotype (Matsuzaki et al., 2018; Liguore et al., 2019). These observations suggest that while being powerful tools to achieve genetic manipulations in the rodent brain (*e.g*. gene replacement, editing, optogenetics), the first generation of PHP variants are unlikely to be used in clinic. This being said, Kumar and colleagues performed a multiplexed screening including a selection step in various mouse strains and they isolated a new capsid (i.e., PHP.C2) that induced good brain transduction even in genetic backgrounds expressing low levels of Ly6a. Yet, this novel variant needs to be tested in other species as well as in disease models in order to determine its potential transferability to humans.

This strategy of capsid engineering does not offer infinite possibilities and because of the shortage in AAV capsids, alternative approaches must be developed in order to overcome the limitations of these novel vectors in particular regarding their use in clinic. Capsid engineering also goes along with changes in the selectivity of tissue infection and cellular tropism. While the PHP.B serotype retains a transduction of peripheral tissues similar to that of the AAV9 parental capsid, the improved brain penetrance of the PHP.eB vector is at the expense of peripheral organs (Chan et al., 2017). This makes the PHP.eB serotype an interesting tool for conditions with dominant CNS pathology but less attractive for multisystem diseases.

Also, despite PHP vectors showed great efficacy in preclinical disease models, they are unlikely to be a solution for all brain disorders. Their region-dependent infectivity as well as their heterogenous tropism for neural cells represents a limitation for some diseases where specific cell types are affected (Deverman et al., 2016; Chan et al., 2017; Challis et al., 2019). This is for instance the case for multiple system atrophy in which alpha-synuclein-rich aggregates accumulate in oligodendrocytes, a cellular population that is poorly infected by these vectors in comparison to neurons and astrocytes (Deverman et al., 2016). Consequently, a “one-size-fits-all” strategy is unlikely to work and similar to the development of personalized medicine, some pathologies may require tailor-made vector constructs with features driven by the disease characteristics.

In summary, recent efforts in designing brain-penetrant AAV-capsids have made an important step forward by generating several tools. Their valuable features have already been challenged in animal models of severe diseases and they demonstrated remarkable therapeutic effects. Regardless of their applicability in a clinical setting, these emerging tools offer a unique opportunity to provide the proof-of-concept that many genetic pediatric diseases with CNS pathology could be rescued by gene delivery when suitable viral vectors will be available. This important piece of information brings new hope for patients affected by these conditions.

## Author Contributions

All authors contributed to the preparation and writing of this paper and agreed on its final version.

## Conflict of Interest

The authors declare that the research was conducted in the absence of any commercial or financial relationships that could be construed as a potential conflict of interest.
